# The Glittre-ADL test reflects functional performance measured by physical activities of daily living in patients with chronic obstructive pulmonary disease

**DOI:** 10.1590/bjpt-rbf.2014.0155

**Published:** 2016-04-08

**Authors:** Manuela Karloh, Cintia L. P. Araujo, Aline A. Gulart, Cardine M. Reis, Leila J. M. Steidle, Anamaria F. Mayer

**Affiliations:** 1 Núcleo de Assistência, Ensino e Pesquisa em Reabilitação Pulmonar (NuReab), Universidade do Estado de Santa Catarina (UDESC), Florianópolis, SC, Brazil; 2 Programa de Pós-graduação em Ciências do Movimento Humano, UDESC, Florianópolis, SC, Brazil; 3 Programa de Pós-graduação em Fisioterapia, UDESC, Florianópolis, SC, Brazil; 4 Curso de Medicina, Departamento de Clínica Médica, Centro de Ciências da Saúde, Universidade Federal de Santa Catarina (UFSC), Florianópolis, SC, Brazil; 5 Departamento de Fisioterapia, UDESC, Florianópolis, SC, Brazil

**Keywords:** activities of daily living, accelerometry, outcome assessment, chronic obstructive pulmonary disease, physical therapy specialty

## Abstract

**Background:**

The Glittre-ADL test (TGlittre) is a valid and reliable test for the evaluation of functional capacity and involves multiple physical activities of daily living (PADL), which are known to be troublesome to patients with Chronic Obstructive Pulmonary Disease (COPD). However, it is still unknown if this test is also able to reflect the functional performance of patients with COPD.

**Objective:**

To investigate whether the TGlittre reflects the functional performance of COPD patients and whether the necessary time to complete the TGlittre and the PADL varies according to disease severity.

**Method:**

Thirty-eight patients with COPD (age 65, SD=7 years; forced expiratory volume in the first second 41.3, SD=15.2% predicted) underwent anthropometric and lung function assessments and were submitted to the TGlittre and PADL measurement.

**Results:**

TGlittre performance correlated significantly (p<0.05) with PADL variables, such as time sitting (r=0.50), walking (r=-0.46), number of steps taken (r=–0.53), walking movement intensity (r=–0.66), walking energy expenditure (r=-0.50), and total energy expenditure (r=–0.33). TGlittre performance was not significantly different in patients among the Global Initiative for COPD (GOLD) spirometric stages, but walking and sitting time were significantly lower and greater, respectively, in severe and very severe patients compared to those with moderate disease (p<0.05).

**Conclusion:**

The performance on the TGlittre correlates with walking and sitting time and other real life PADL measurements. The severity of the disease is associated with the differences in the level of physical activity in daily life more than in functional capacity.

## Bullet points

- The Glittre-ADL test (TGlittre) reflects the functional status of patients with COPD.
- The TGlittre is a simple and feasible test suitable for functional assessment in clinical practice.
- The TGlittre can be an easy-to-apply method of assessing PADL limitation in COPD.

## Introduction

The deterioration of functional status is an important manifestation in patients with chronic obstructive pulmonary disease (COPD)^1^ ^-^ ^3^. It is characterized by limitations in activities of daily living (ADL) and reduction in physical activities of daily life (PADL) and physical activity level^4^ ^,^ ^5^, the last being considered the strongest predictor of all-cause mortality in patients with COPD^6^. As defined by Leidy^7^, functional status is a multidimensional concept characterizing the ability that a person has to provide for the necessities of life and involves four constructs: functional capacity, performance, reserve, and capacity utilization. These constructs are distinct, but related and should be considered when selecting tools for functional outcome assessments^7^. Functional capacity is the maximum potential to perform activities, while functional performance refers to the day-to-day activities that people actually choose and need to do during their normal routines depending on the limits imposed by their functional capacity^8^. Although daily performance is limited by functional capacity, people generally perform fewer PADL than they actually can do and the intensity of the activities that they perform is below their functional capacity^9^. Kocks et al.^8^ report that functional capacity may be the most important factor for research purposes since it is directly related to the effects of the intervention. On the other hand, they consider functional performance more relevant for clinical management because it reflects the patient’s experiences.

Based on the need to chose an outcome according to the main purpose (research or clinical) and since the improvement of functional status is one of the major goals of pulmonary rehabilitation programs^1^, the evaluation of their constructs and the choice of instruments should be done carefully because they can improve the likelihood of detecting true treatment effects in both research and clinical settings. Functional performance can be evaluated by direct video observation, movement monitors, or questionnaires^8^. Direct observation is the gold-standard, however the process is time-consuming, intrusive, and unsuitable for large populations^10^. Movement monitors are accurate tools that have been validated for functional performance assessment^4^ ^,^ ^10^ ^,^ ^11^, but their higher cost makes their use less feasible in clinical settings. Questionnaires are low-cost and easy to apply but they can be easily influenced by psychological factors or cognitive deficits, since they do not objectively evaluate patient limitations^12^. Given the difficulty, higher cost, and lower viability of these tools, instruments to measure functional capacity objectively could be used for this purpose. However, they must be able to represent the functional performance of patients with COPD and reflect real-life situations more reliably.

The Glittre ADL-test (TGlittre) is a performance-based test that was developed to reflect real-life situations better, thus improving the assessment of functional capacity of stable^12^ or hospitalized^13^ COPD patients and providing additional information about their ability to perform PADL. It is especially effective in more severe patients^12^ because it involves common activities essential to everyday life and known to be troublesome for them. However, a recent review of available methods of functional status measurements in COPD categorized the TGlittre as being a test for both capacity and performance evaluation^8^ given that it is a multiple PADL task-test. Nevertheless, it is not yet known if TGlittre is actually able to reflect functional performance in COPD patients. This investigation is important because the TGlittre is a simple and feasible test suitable for clinical practice, health services, and research and it is easy to administer and more accessible than movement monitors.

The present study aimed to investigate whether the TGlittre reflects the functional performance assessed by PADL monitoring in COPD patients. A second aim was to investigate whether the time necessary to complete the test and the PADL varies according to the Global Initiative for Chronic Obstructive Lung Disease (GOLD)^3^ spirometric classification stages.

## Method

### Subjects

The study included COPD patients with GOLD stages 2-4^3^ who were clinically stable in the four weeks prior to the study protocol and whose age was ≥40 years old. Exclusion criteria were long-term oxygen therapy, current smoking, pulmonary disease other than COPD, and any comorbidities that could compromise their ability to perform or understand any of the evaluations in the study. Clinically stable patients with medical diagnosis of COPD were recruited from March 2010 to March 2012 from the pulmonology outpatient units of local public hospitals and private clinics in Florianópolis, SC, Brazil. None of the patients had ever been included in pulmonary rehabilitation programs.

### Study design

This is a cross-sectional observational study. Anthropometric and lung function assessments were carried out in all subjects. On the same day, the patients underwent a TGlittre familiarization trial. On a different day within a week from the familiarization trial, one TGlittre trial was performed. The patients’ PADL were also monitored for two consecutive days starting the day after the TGlittre trial. A symptom questionnaire was applied to determine clinical stability between the evaluations^14^. The study was approved by the Ethics Committee of Universidade do Estado de Santa Catarina (UDESC), Florianópolis, SC, Brazil (approval number 175.484) and all participants signed a written informed consent form.

### Pulmonary function assessment

Lung function was assessed using an EasyOne spirometer (NDD Medical Technologies, Zurich, Switzerland), whose calibration was checked before each evaluation. Spirometry was performed in accordance with the American Thoracic Society/European Respiratory Society standards^15^. Forced vital capacity (FVC) and forced expiratory volume in one second (FEV_1_) were measured in liters and percentage of the predicted value (%pred). The predicted values were calculated from the equations proposed by Pereira et al.^16^.

### Physical activity in daily life

In order to quantify PADL, the patients were monitored with an accelerometer-based activity monitor (DynaPort MiniMod; McRoberts BV, The Hague, The Netherlands) for 12 hours on two consecutive weekdays, beginning immediately after awakening. All subjects were carefully instructed on how the device should be positioned and received a manual with clear instructions. In addition, the patients were instructed to make no changes to their routine of daily activities while wearing the device^4^. We measured the time spent sitting, lying, standing, and walking, the movement intensity during walking, the energy expenditure during these positions/movements, and the number of steps. Mira2 software (McRoberts BV, The Hague, The Netherlands) was used to read and process the accelerometer data.

### Glittre ADL-test

The TGlittre consists of completing a circuit while carrying a weighted backpack (2.5 Kg for women, 5.0 Kg for men). The 10-m long circuit is laid out as follows: from a sitting position, the patient stands up and walks along a flat course, traversing a two-step staircase at the midpoint (17 cm high × 27 cm deep each step); after completing the second half, the patient moves three 1 Kg objects from a shelf at shoulder height to another one at waist height and then to the floor; then, the patient returns the objects to the bottom shelf and finally to the top shelf again; then, the patient walks back the way he came, climbing and descending the stairs, until reaching the starting point (chair) again; sits down and immediately begins the next lap. Patients were instructed to complete five laps on this circuit in the shortest time possible^12^. Heart rate, peripheral oxygen saturation using a pulse oximeter, and dyspnea assessed by the Modified Borg Scale^17^ were measured at the beginning and end of each lap and at the end of the test.

### Sample size calculation

The sample size was calculated to answer the primary aim of the study and it was based on the correlation between physical activity level and six-minute walk test (6MWT) distance (r=0.46; p<0.001) found by Watz et al.^18^. Based on their results^18^ and using a two-sided alpha=0.05 and a power of 80%, the estimated number of patients necessary to complete the present study was 36. Considering a drop-out rate based on a pilot study of our own laboratory (unpublished data), the final sample size was 38 patients.

### Statistical analysis

The data were reported as mean and standard deviation. The Shapiro-Wilk test was used to analyze data normality. The Spearman correlation coefficient was calculated to test the relationship between time spent in the TGlittre and PADL for the entire group. The Pearson or Spearman correlation coefficient was used, according to data normality, to determine the correlations between TGlittre performance and PADL variables in each GOLD stage group. The strength of the correlations was defined according to Munro's categories: weak 0.26-0.49, moderate 0.50-0.69, strong 0.70-0.89, and very strong 0.90-1.00^19^. To compare data between GOLD stages, one-way ANOVA and the Tukey test were applied. Statistical significance was set at p<0.05. Data analysis was performed with SPSS 20.0 (IBM SPSS Statistics, IBM Corp, Somers, NY, USA) and the graphs were produced in Graphpad Prism 5.0 (GraphPad Software Inc., San Diego, CA, USA).

## Results

Twelve out of 55 invited participants declined to take part in the study and 38 completed the study (22 men). Five patients were excluded: three due to inability to perform the proposed tests, one for returning to smoking during the protocol, and one for being diagnosed with bronchiectasis during the study.

The characteristics of the subjects are presented in Table 1. Subject age ranged from 51 to 79 years, and FEV_1_ ranged from 15 to 69% of predicted (41.3, SD=15.2%pred). The time necessary to complete the TGlittre was 4.69, SD=1.28 min (range from 3.15 to 9.50 min) (Table 1).

**Table 1:** Characteristics of the study group.

	Group (n=38)	GOLD 2 (n=11)	GOLD 3 (n=17)	GOLD 4 (n=10)
Age, years	65 (63-68)	61 (57-66)	67 (63-71)	66 (62-70)
BMI	27.7 (26.1-29.2)	28.5 (25.5-31.5)	28.7 (26.3-31.0)	25.0 (21.4-28.7)
TGlittre, min	4.69 (4.27-5.11)	4.02 (3.52-4.53)	4.87 (4.27-5.48)	5.12 (3.91-6.92)
Time sitting, min	381 (351-412)	316 (273-359)	406 (360-452)^*^	410 (341-480)^*^
Time lying, min	77.1 (53.3-101)	108 (60.1-156)	58.0 (25.6-90.4)	75.2 (16.2-134)
Time standing, min	155 (140-171)	167 (127-208)	157 (137-178)	140 (108-170)
Time walking, min	81.1 (68.1-94.0)	108 (81.3-134)	74.8 (56.3-93.3)^*^	62.4 (39.6-85.2)^*^
Number of steps taken	6557 (5496-7619)	8605 (6467-10743)	5904 (4194-7613)^*^	5415 (3883-6947)^*^
Movement intensity, m/s^2^	1.78 (1.70-1.87)	2.01 (1.88-2.14)	1.70 (1.57-1.81)^*^	1.60 (1.53-1.85)^*^
Walking energy expenditure, kcal	386 (314-459)	526 (366-686)	341 (233-450)^*^	309 (198-421)^*^
Total energy expenditure, kcal	1392 (1283-1501)	1563 (1318-1808)	1367 (1209-1525)	1246 (1051-1443)

Mean (95% Confidence Interval); BMI: body mass index; TGlittre (min): time, in minutes, spent in the Glittre-ADL test.

* p<0.05 vs GOLD II.

The patients remained for more than half of the monitored time, 53% (SD=13%) in the sitting position, 11% (SD=10%) in the lying position, 22% (SD=7%) standing, only 11% (SD=6%) walking, and 3.40% (SD=1.80%) in other active positions. The time spent by patients in each PADL can be seen in Table 1. The sample walked mainly for short periods of time lasting less than one min. Four patients walked continuously for longer than 10 min, five between 10 and 20 min and two between 20 and 30 min. Only one patient underwent a period of continuous walking longer than 30 min. The patients spent 617 (SD=46) min/day in sedentary activities according to the metabolic equivalent of task or MET (<3 METs), 98.6 (SD=45.2) min/day in moderate activity (3-6 METs), and only 4.65 (SD=6.63) min/day in vigorous activity (6-9 METs).

**Figure 1:**
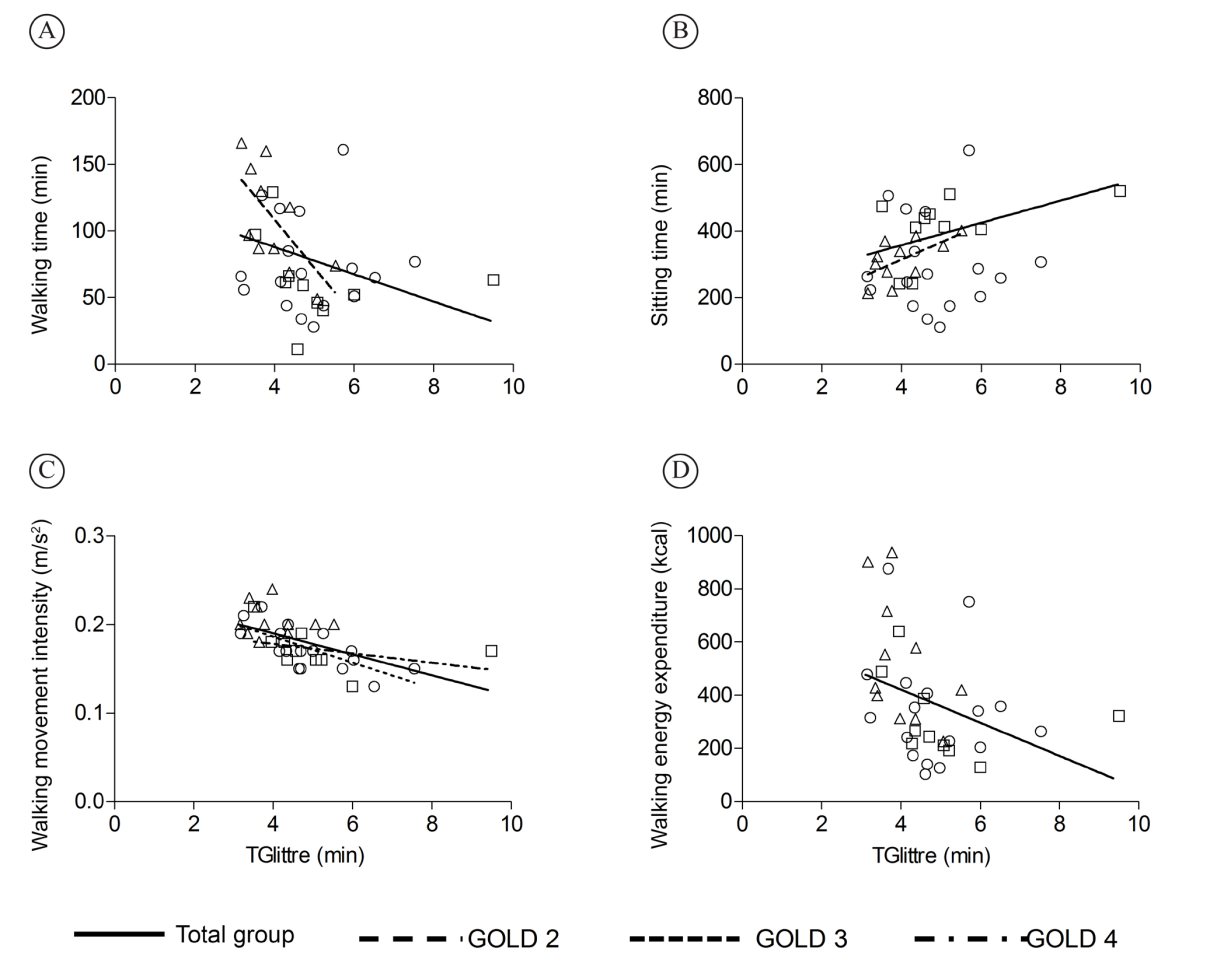
Correlations between performance in the Glittre-ADL test (TGlittre) and physical activities of daily living: (A) r=-0.46; (B) r=0.50; (C) r=-0.66; (D) r=-0.50 (p<0.05 for all); (∆=GOLD 2, ○=GOLD 3, and □=GOLD 4).

**Figure 2:**
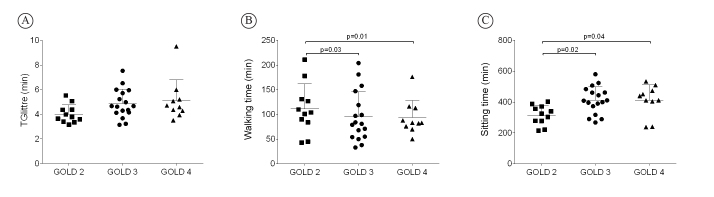
Differences in TGlittre time (A), walking time (B), and sitting time (C) among the GOLD stages.

TGlittre performance correlated with several PADL variables, such as time sitting (r=0.50; p<0.01), walking (r=-0.46; p<0.01), the number of steps taken (r=–0.53; p<0.01), walking movement intensity (r=–0.66; p<0.01), walking energy expenditure (r=–0.50; p<0.01), and total energy expenditure (r=–0.33; p=0.04) (Figure 1A-D). The performance on this test significantly correlated with walking (r=–0.69; p=0.02) and sitting time (r=0.61; p=0.04) in the GOLD 2 group; with movement intensity during walking (r=–0.73; p<0.01) in the GOLD 3 group; and with the number of steps taken (r=–0.65; p=0.04) and walking movement intensity (r=–0.70; p=0.02) in the GOLD 4 group. TGlittre performance was not significantly different among patients when they were divided according to GOLD (p=0.08) (Figure 2A), but the time walking (Figure 2B) and sitting (Figure 2C) was significantly different between severe/very severe patients and those with moderate disease (p<0.05; Table 1).

## Discussion

The present study aimed to investigate whether the TGlittre reflects the functional performance of COPD patients assessed by PADL monitoring and to investigate whether the time necessary to complete the TGlittre and the PADL varies according to GOLD spirometric stages. This study demonstrated, in a sample of patients with moderate to very severe COPD, that time spent in the TGlittre correlates with real-life measurements of functional performance by a motion sensor, such as time walking and sitting, the number of steps taken, energy expenditure, and movement intensity during walking. The correlation between PADL and functional capacity according to the 6MWT has been previously demonstrated and varies from 0.42 to 0.76^4^ ^,^ ^18^ ^,^ ^20^ ^,^ ^21^. The findings of this study confirm that PADL, i.e. the functional performance of patients with COPD, are best predicted by global tests that involve various components^4^. However, this is the first time that an association has been demonstrated between PADL and functional capacity according to the TGlittre, besides confirming the test’s ability to assess the functional limitations related to COPD^12^. These findings are very similar to those previously described for the 6MWT^4^ ^,^ ^18^ ^,^ ^20^ ^,^ ^21^. Moreover, a novel association between TGlittre performance and time spent in inactive postures, such as sitting, was found in the present study. This association was also found in less severe patients when the sample was divided according to GOLD stage. This may imply that the amount of time spent in inactive postures could be used to determine functional limitation in less-impaired COPD patients. In more severe patients (GOLD 3 and 4), active postures (walking time, the number of steps taken, and movement intensity during walking) are better associated with functional capacity. The TGlittre also involves periods with no work (e.g. sitting) and relatively static postures (e.g. standing), which would closely reflect the inactive postures during PADL. It was also shown that movement intensity seems to be better associated with the TGlittre than walking time and energy expenditure. We could infer that before reducing the walking time, the patients initially slow their walking. Then with disease progression, it seems that they reduce the walking time and perform it even more slowly. Thus, the reduction in the movement intensity could be an earlier sign of the disease’s impact on the functional status of patients with COPD than the reduction in walking time in daily life.

The TGlittre was developed to simulate daily activities in a field test so as to better reflect the real-life situations of these patients and can also differentiate the functional capacity of these patients from aged-matched healthy subjects^22^. The selection of activities for this test was based on the modified version of the Pulmonary Function Status and Dyspnea Questionnaire^23^ and the London Chest Activity of Daily Living Scale^24^, whose items are known to cause limitation in patients with COPD. In a recent systematic review regarding the measurements of PADL in COPD, Janaudis-Ferreira et al.^25^ found that the TGlittre was one of three performance-based tests available (i.e. tests that include more than three types of PADL) and the only one specifically developed for patients with COPD. According to these authors, performance-based tests are ideal to detect the patients’ actual performance even though they may not reflect a full spectrum of PADL performed by patients. Also, the study above showed that among the 27 instruments included, the TGlittre was one of the only five that evaluated responsiveness and one of the two that were directly or indirectly associated with healthcare utilization. After the TGlittre validation study was conducted, it was demonstrated that there is a positive correlation between performance results in the different activities in the test (0.62<r>0.95, p<0.0001)^26^. Cavalheri et al.^26^ demonstrated that climbing up and down stairs was the most demanding activity, with higher energy expenditure, heart rate, sensation of dyspnea and fatigue, while moving the objects on the shelf required less energy. There were no differences in energy expenditure between walking activities with or without a backpack and sitting and rising from a chair^26^.

Some of the TGlittre activities can be directly identified in physical ADL monitoring. For example, the TGlittre sitting activity might correspond to sitting time during accelerometer monitoring, walking on a flat surface during TGlittre might correspond to the time spent walking in PADL, and moving objects between the shelves might correspond to the time spent standing in PADL. Climbing up and down stairs is the only activity not easily identified by the motion sensor used in this study, since displacement is evaluated only by the walking time and number of steps. However, since this activity generates the highest energy expenditure in patients with COPD^26^, the time that patients spend climbing up and down stairs in their daily routine could be inferred from higher values of energy expenditure recorded by the monitor during walking and more accurately so when matched with their activity diary. Another important finding in this study was that only the PADL (walking time, sitting time, and movement intensity during walking) varied among the GOLD stages; the TGlittre performance did not. Although there were no differences in TGlittre performance, the standard deviation of the measure was higher in GOLD 3 and 4 than in GOLD 2, showing that the variance of time to complete the TGlittre is more pronounced in those patients. In addition, it could explain the lack of statistical difference among GOLD stages. Previous studies have shown PADL differences in different severities of the disease, such as the time spent standing^4^, the number of steps taken per day, and the time spent in activities with energy expenditure exceeding three METs^18^. Unlike the present study, in which no difference could be found in functional capacity between GOLD stages, Watz et al.^18^ found differences in the 6MWT distance in patients with GOLD stage 1-2 and 3-4 as well as differences in the level of physical activity. This means that, in the present sample, functional performance seemed to be more sensitive for differentiating patients in terms of disease severity than functional capacity. These findings reaffirm the importance of not only evaluating the functional capacity of patients with COPD but also incorporating the assessment of functional performance in the routines of pulmonary rehabilitation programs. Indeed, physical activity level is considered the best predictor of all-cause mortality in these patients, more so than 6MWT distance and disease severity^6^.

As previously described^4^ ^,^ ^20^, it was also observed in this study that patients with COPD spent most of their time in inactive postures. These results corroborate the sedentary lifestyle adopted by patients with COPD due to the disease’s numerous consequences, such as airflow obstruction, dynamic hyperinflation, air trapping, and reduced peripheral muscle strength, which lead to reduced functional and exercise capacity, among other negative effects^3^ ^,^ ^27^ ^-^ ^29^. Besides the evident inactive profile of the studied group, it is worth noting that the patients from the present sample were less inactive than the patients from some studies previously published^4^ ^,^ ^5^ ^,^ ^20^ ^,^ ^30^, but the reasons for that have not been investigated in this study.

The inactivity of patients with COPD becomes more obvious in light of American College of Sports Medicine (ACSM) recommendations^31^, which suggest 30 minutes of daily physical activity of moderate intensity, such as walking, for an individual to be considered physically active. Thus, another important finding in this sample is that although the mean time of walking and moderate activity was over 30 minutes (81.1, SD=39.3 and 98.6, SD=45.2 min/day), only one patient superseded ACMS recommendations with continuous walking and another met the recommendations in two 15 min walking sessions. This same patient had the fourth best performance in the TGlittre, completing the test in 3.52 min, which was lower than the mean group time. Most patients showed a pattern of fragmented walking in short periods lasting less than a minute. This pattern of short periods of activity is already known in COPD patients, even after a pulmonary rehabilitation program. Patients become more active by performing a greater number of short periods of walking^30^.

To our knowledge, this is the first study demonstrating the relationship between a specific multiple-task PADL test and real-life PADL measurement. This adds value and usefulness to the TGlittre, which provides complementary information in the functional assessment of COPD patients by involving four activities other than walking. The TGlittre can be very useful in clinical practice to assess not only functional capacity but also functional performance of patients with COPD. Furthermore, the power analysis based on the correlation between the main variables showed a power of at least 80% to detect a medium or strong correlation coefficient.

Some limitations should be made clear. Although the sample size was sufficient to answer our primary objective, we did not estimate a sample size capable of comparing PADL (power of 90%) and time taken to complete the TGlittre (power of 48%) between GOLD stages. The results, however, were consistent among these patients and thus seem to be representative for this group. Nevertheless, further research should be conducted to confirm the finding that functional performance might be more sensitive than functional capacity to differentiate patients with COPD according to disease severity. In addition, the choice for two days of monitoring could be considered a potential bias for this measurement. However Pitta et al.^4^, have showed that only two consecutive week days of assessment are necessary to achieve a reliable PADL measurement.

## Conclusions

This study demonstrated that performance on the TGlittre correlates with walking and sitting time, the number of steps taken, energy expenditure, and movement intensity during walking in real life when monitored by a motion sensor. The severity of the disease seems to be associated with differences in the level of physical activity in daily life more than in functional capacity for performing PADL.
